# Chronic and Recurrent Herpes Zoster Ophthalmicus

**DOI:** 10.3390/medicina57100999

**Published:** 2021-09-22

**Authors:** Soo Min Lee, Jisang Han, Chan Min Yang, Chul Young Choi, Ramin Khoramnia, Tae-Young Chung, Dong Hui Lim

**Affiliations:** 1 Nunemiso Eye Center, Seoul 06241, Korea; bessential@naver.com; 2 Department of Ophthalmology, Samsung Medical Center, Sungkyunkwan University School of Medicine, Seoul 06351, Korea; ophycm@gmail.com; 3 Department of Ophthalmology, Kangbuk Samsung Hospital, Sungkyunkwan University School of Medicine, Seoul 03181, Korea; jisang.han@samsung.com (J.H.); chulyoung.choi@samsung.com (C.Y.C.); 4 Department of Ophthalmology, University of Heidelberg, 69102 Heidelberg, Germany; Ramin.Khoramnia@med.uni-heidelberg.de; 5 Samsung Advanced Institute for Health Sciences and Technology, Sungkyunkwan University, Seoul 06355, Korea

**Keywords:** herpes zoster ophthalmicus, epidemiology, frequency

## Abstract

*Background and Objectives*: This study sought to investigate the natural course, the chronicity and recurrence rate, and the risk factors of chronic and recurrent herpes zoster ophthalmicus (HZO). We also evaluated the effects of long-term treatment for HZO. *Materials and Methods*: Patients diagnosed and treated for HZO were included in the retrospective medical chart review. Multivariable-adjusted logistic and Cox regression models were used to show risk factors for chronic and recurrent HZO along with hazard ratios (HRs) and 95% confidence intervals (CIs). *Results*: Among a total 130 of HZO patients, 31 patients (23.85%) had chronic disease and 19 patients (14.62%) had recurrent disease. The rate of chronic disease was higher in HZO with conjunctivitis, epithelial keratitis, and stromal keratitis. The recurrence rate increased in patients with chronic HZO (HR: 34.4, 95% CI: 3.6–324.6), epithelial keratitis (HR: 5.5, 95% CI: 1.3–30.0), stromal keratitis (HR: 18.8, 95% CI: 3.0–120.8), and increased intraocular pressure (IOP) (HR: 7.3, 95% CI: 1.6–33.2). Length of systemic antiviral therapy and anti-inflammatory eyedrop treatment were not associated with recurrent HZO (*p* = 0.847 and *p* = 0.660, respectively). The most common ocular manifestation for recurrent HZO was stromal keratitis. *Conclusions*: This study demonstrated a considerable frequency of chronic and recurrent HZO. Chronic HZO in the form of epithelial or stromal keratitis with increased IOP provoked a significant rise in the risk of recurrence.

## 1. Introduction

Herpes zoster (HZ) is defined as the reactivation of the latent varicella zoster virus (VZV) in people who have had chickenpox (varicella), the primary infection caused by VZV. After the primary infection, the virus is latent for a period of time in the sensory ganglia. If and when the latent virus is reactivated, rashes and vesicles occur along the affected dermatomal distribution, with accompanying pain [1]. It is believed that viral reactivation is a result of declining cell-mediated immunity, which can be associated with aging, impaired immunity, trauma, and psychological stress [2]. Although the prevalence of HZ has not been clearly documented, it can be predicted that the prevalence of HZ will increase with the increasing age of the population and immune suppression therapy considering the risk factors of HZ [3,4,5]. It is estimated that one million episodes occur each year in the United States alone [5,6,7].

HZ ophthalmicus (HZO) is defined as HZ involvement of the first branch of the trigeminal nerve ganglion with ocular involvement. HZO accounts for 10% to 20% of all HZ cases [8]. Decreased cellular immunity, advancing age, immunosuppressive medication, and primary infection with the HZ virus in infancy have been found to increase the risk of HZO [8,9,10,11].

The ocular manifestations of HZO include conjunctivitis, epithelial keratitis including punctate erosion or pseudodendrites, stromal and endothelial keratitis, acute and postherpetic neuralgia, vesicular dermatitis and preseptalcellulitis, orbital cellulitis, uveitis, glaucoma, and optic neuritis [11,12].

Although long-term or recurrent HZO may persist in some patients, there is not much known about the prevalence and treatment regimen for such. Thus, this study aimed to identify the epidemiology of HZO in Koreans and the risk factors for prolonged or recurrent HZO. We also aimed to elucidate the relationship between treatment duration and the recurrence of HZO.

## 2. Methods

This is a retrospective cohort study of patients diagnosed with HZO using electronic medical records from Samsung Medical Center in Seoul, Korea, between 26 July 2009 and 31 May 2016. The study was conducted in accordance with the tenets of the Declaration of Helsinki and approved by Samsung Medical Center Institutional Review Board.

We reviewed all patients’ charts who received a diagnostic code of HZ and underwent antiviral treatment in all departments of the hospital. Korean Standard Classification of Disease (KCD) codes B02.0 to B02.9, which are associated with zoster, were used to screen for HZO. Patients with HZO were identified from among the HZ patients. The medical records of potential patients with HZO were reviewed to confirm the diagnosis of HZO. The sex, age, medical history, ophthalmologic clinical findings, treatment period, and immune status of all patients were investigated. Treatment period refers to the duration of antiviral or anti-inflammatory treatment. Primary treatment duration means the treatment duration for the first disease attack. We defined patients with an immunocompromised state as follows: patients who underwent systemic chemotherapy within six months prior to the diagnosis of HZO, who had actively treated malignancies, who had end-stage renal disease, who had autoimmune diseases treated with immunosuppressive drugs, and/or who had organ transplantation. None of the patients had an infection with the human immunodeficiency virus, and none had congenital immunodeficiency disease.

Ophthalmologic clinical findings were classified according to the ophthalmologist’s medical records in terms of conjunctivitis, epithelial keratitis including pseudodendritic keratitis, stromal keratitis, endothelial keratitis, uveitis, glaucoma, and optic neuritis. All patients underwent measurements of visual acuity and intraocular pressure (IOP) and microscopic slit lamp examination at every visit.

The study participants were divided into two groups based on the duration of disease. Chronic HZO was defined as active disease requiring antiviral or anti-inflammatory treatment persisting for more than 60 days. Therefore, acute HZO was defined as the termination of treatment within 60 days. In addition, we defined recurrent HZO as a case in which the clinical findings completely disappeared and the ocular involvement recurred any time after termination of treatment.

Baseline characteristics of the study population and clinical findings were presented as numbers with percentages (%) for categorical variables or mean values with standard deviations for continuous variables. The chi-square test was used to calculate the statistical difference between categorical variables. Univariable and multivariable-adjusted logistic and Cox regression models were used to show risk factors for chronic and recurrent HZO along with hazard ratios (HRs) and 95% confidence intervals (CIs). Kaplan–Meier survival analysis was employed to characterize the HZO recurrence-free survival curve, and *p* values of less than 0.05 were considered to be statistically significant. Statistical analyses were performed using SAS version 9.4 software program (SAS Institute, Cary, NC, USA).

## 3. Results

### 3.1. Baseline Characteristics

**Figure 1:**
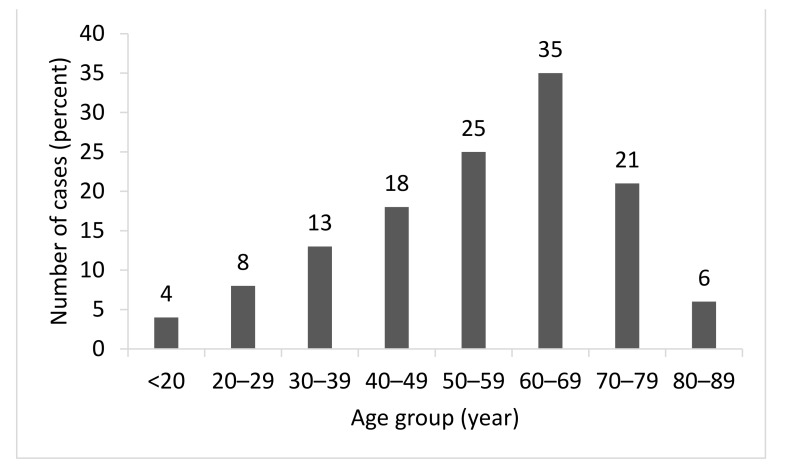
Age distribution of patients at first diagnosis of HZO. The number of patients for each age group is indicated in each bar graph. There were significant differences between the various age groups (*p* < 0.0001 *). * Chi-squared test. Abbreviations: HZO = herpes zoster ophthalmicus.

A total of 1875 patients were found to have been diagnosed with HZ during the study period, with 163 having been specifically diagnosed with HZO. Through detailed medical chart review, we excluded 33 patients who were misdiagnosed as having HZO, who had undergone ophthalmic surgery less than a year before the manifestation of HZO, or who had a history of ophthalmic disease that could affect the development and progress of the disease; thus, 130 patients were included in the final analysis. Demographics and characteristics of the study population are summarized in Table 1. The male to female ratio was 1.03. No significant sex difference was observed (*p* = 0.8608). In male patients, 12 had chronic and five had relapsed HZO; among the female patients, 19 had chronic and 14 had relapsed HZO. The mean age of the study participants was 55.18 ± 17.66 years (range: 11–89 years). The majority of patients were immunocompetent (89.23%). The mean treatment duration was 82.22 ± 199.56 days (range: 3–1575 days), and the median value of treatment duration was 11 days. The distribution of HZO patients according to their age is shown in Figure 1. Of all of the HZO patients, most were in their 60s, followed by in their 50s and 70s (35%, 25%, and 21%, respectively).

### 3.2. Clinical Manifestations

Ocular manifestations were present in 104 (80%) patients. Twenty-six (20%) patients had skin rash only in the V1 dermatome. The distribution of HZO-associated ocular manifestations is summarized in Table 2. The most common ocular manifestations were epithelial keratitis, followed by conjunctivitis/episcleritis and endothelial keratitis (46.15%, 37.69%, and 11.54%, respectively). Stromal keratitis was present in 10 patients (7.69%) and uveitis was present in eight patients (6.15%). Five patients presented with increased IOP in the first month and another five patients presented with increased IOP in the second month after beginning treatment. There were two patients with cranial nerve palsy.

### 3.3. Clinical Course

#### 3.3.1. Chronic Disease

Of the 130 patients with HZO, 99 (76.15%) had an acute course and 31 (23.85%) had a chronic course. The mean treatment duration of acute-course patients was 12.78 ± 10.27 days (range: 3–50 days) and the median treatment duration was eight days. In comparison, the mean treatment duration of chronic-course patients was 304.00 ± 322.74 days (range: 66–1575 days) and the median treatment duration was 210 days. In univariate logistic regression analysis, age, sex, conjunctivitis, epithelial keratitis, stromal keratitis, corneal endotheliitis, uveitis, and increased IOP were identified as possible risk factors with *p* values of less than 0.2 for chronic HZO (Table 3). In multivariate logistic regression analysis, conjunctivitis, epithelial keratitis, and stromal keratitis were associated with chronic HZO (*p* = 0.0154, *p* = 0.0005, and *p* = 0.0005, respectively).

#### 3.3.2. Recurrent Disease

Of the 130 patients with HZO, 19 (14.62%) had recurrent HZO. Of the 19 cases of recurrent HZO, 18 also presented with chronic disease. The mean time from the end of treatment to recurrence was 6.24 ± 8.76 months (range: 0.36–35.04 months). The median time of recurrent HZO treatment was 8.93 months. In univariate analysis, sex, conjunctivitis, epithelial keratitis, stromal keratitis, corneal endotheliitis, uveitis, increased IOP, primary treatment duration, and chronic HZO were identified as possible risk factors for recurrent HZO with *p* values of less than 0.2 (Table 4). Through multivariate analysis using a Cox proportional hazards model, we determined that epithelial keratitis, stromal keratitis, increased IOP, and chronic HZO (*p* = 0.0465, *p* = 0.0020, *p* = 0.0101, and *p* = 0.0020, respectively) increased the risk of recurrent HZO. Conversely, gender, age, and immune status did not increase the risk of chronic or recurrent HZO.

The distribution of recurrent HZO-associated ocular manifestations is summarized in Table 5. The most common recurrent HZ ocular manifestation was stromal keratitis, followed by epithelial keratitis, endotheliitis, and conjunctivitis/scleritis (50.00%, 29.17%, 12.50%, and 8.33%, respectively). Of the 12 recurrent herpes zoster patients with stromal keratitis, three patients did not have stromal keratitis at the initial visit.

## 4. Discussion

**Figure 2:**
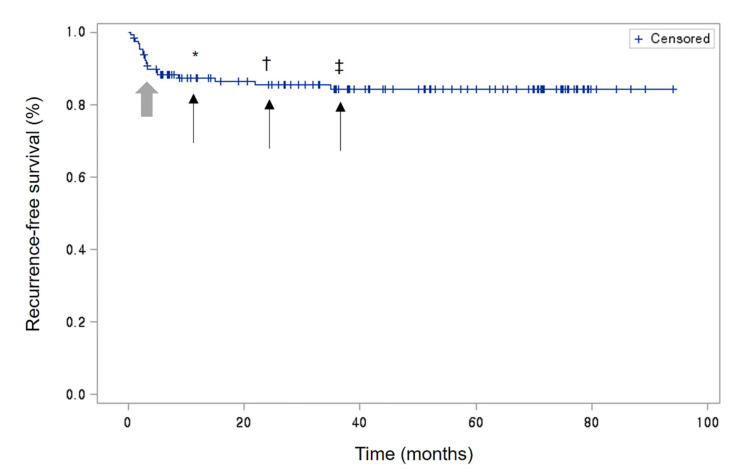
Kaplan–Meier survival analysis demonstrating cumulative recurrence-free survival over time. Within one year, 89.5% of all events recurred; the curved point (bold arrow) of the graph corresponds to 8.6 months. * One-year survival was 87.44%. † Two-year survival was 85.5%. ‡ Three-year survival was 84.38%.

This study identified a considerable frequency of chronic (23.8%) and recurrent (14.6%) HZO. In the risk factor analysis of our study, we found that chronic HZO was associated with conjunctivitis (odds ratio (OR): 8.7; *p* = 0.0154), epithelial keratitis (OR: 41.5; *p* = 0.0005), and stromal keratitis (OR: 46.6; *p* = 0.0005), while recurrent HZO was associated with epithelial keratitis (HR: 5.5; *p* = 0.0465), stromal keratitis (HR: 18.8; *p* = 0.002), increased IOP (HR: 7.3; *p* = 0.010), and chronic HZO (HR: 34.4; *p* = 0.002). Especially, most of the recurrences were seen within 8.6 months after the end of treatment (Figure 2). In the Kaplan–Meier survival analysis demonstrating cumulative recurrence-free survival over time, the overall one-, two-, and three-year recurrence rates were 12.56%, 14.5%, and 15.62%, respectively. Even patients with suspected simple conjunctival involvement can develop a chronic disease, so careful treatment and follow-up are needed. This might be explained by the fact that it is not always easy to distinguish between a simple conjunctivitis and episcleritis or scleritis. Therefore, patients with these risk factors should be treated with consideration of the possibility of chronicity or recurrence of the disease.

In the United States, several studies have demonstrated an increased incidence of HZ [5,13,14,15]. HZ affects approximately 20% to 30% of the general population at some point in their lifetime in the United States [16]. Considering this large prevalence rate, the Centers for Disease Control and Prevention recommended a vaccine (Zostavax^®^ (zoster vaccine live), Merck & Co., Kenilworth, NJ, USA) be given to aged immunocompetent people older than 60 years. HZO accounts for approximately 10% to 20% of all cases of HZ. The spectrum of ocular manifestation of HZO is diverse.

There have been few previous studies conducted to date regarding the recurrence of HZO. To our knowledge, there has only been one investigation published by Tran et al., who evaluated potential risk factors such as demographics, immune status, vaccination status, and ophthalmic involvement for recurrent and chronic HZO using South Florida Veterans Administration Healthcare System (MIAVHS) medical data between 1 January 2010 and 31 December 2014 [17]. Of the 1358 patients with HZ, a total of 90 HZO patients were included in their study. Only increased IOP and uveitis raised the risk of recurrent and chronic disease. Thus, the risk factors for recurrent and chronic disease identified in the study of MIAVHS and in our study are different. However, this may be due to differences in the study populations (e.g., Western vs. Korean); furthermore, while we are a single hospital center, we are a tertiary hospital to which severe cases are referred. Regardless, it is meaningful that both in the previous study and in our assessment, sex, age, and immune status did not increase the risk for a recurrent or chronic HZO.

Of note, there was also an article published regarding the recurrence of HZ, not HZO, by Yawn et al. In Olmsted County, Minnesota, of the 1669 patients with HZ, 105 experienced HZ recurrence between one day and 11.7 years from 1996 to 2001. In this study, recurrence of HZ was defined as a characteristic vesicular rash with pain at three months after the first episode. Episodes of recurrence were more likely to present in individuals with zoster-associated pain of 30 days or longer, immunocompromised status, female sex, and older age (≥50 years) [18]. However, this article differs from our paper because we analyzed the risk factors for HZO, not HZ.

While there has not been enough research studying the long-term disease progress of HZO completed in the past, a large-scale clinical study has been recently started. The Zoster Eye Disease Study (ZEDS) started in the United States in August 2017 to determine whether prolonged suppressive oral antiviral treatment with valacyclovir reduces the complications of HZO.

In our study, the duration of medical treatment did not affect the course of the disease. Endotheliitis and uveitis were not related with chronic and recurrent HZO. We can speculate that endotheliitis or uveitis are not common manifestations of initial attack among recurred or chronic cases. Rather, endotheliitis, uveitis, or increased IOP become more common with prolonged disease course or recurrence. Stromal keratitis and uveitis are known to be the most common manifestation among recurred cases. [19] Because this was a retrospective study, we could not analyze a variety of drugs individually. It is a drawback of retrospective observational study that we could not investigate the exact mechanism between various type of HZO and disease course. Therefore, prospective studies are needed.

In addition, most of the recurrence cases occurred within one year of the initial presentation. Thus, especially if patients have risk factors such as epithelial keratitis, stromal keratitis, or increased IOP, ophthalmologists need to follow up on the progress of the disease for at least one year.

Our study has several limitations. First, it analyzed hospital-based clinical data, so only patients who visited to receive regular care were included in the study. Using data from a single center may cause selection bias and, since our hospital is one of the largest tertiary centers in South Korea, severely affected patients are often referred. Patients who recover quickly or who have no complications may not be treated in a tertiary hospital. Therefore, our chronic disease rate or recurrence rate may be higher than what is typical. Thus, more research is needed to better understand the disease in the entire population. Second, HZO was clinically diagnosed by ophthalmologists and most patients did not undergo laboratory testing, including finding immunoglobulin M antibodies specific to VZV, viral culture, or polymerase chain reaction. In general, the diagnosis of HZO is performed clinically. However, since the clinical findings of HZO are very typical, this clinical diagnostic method does not appear to cause a large bias. Third, we could not always accurately identify whether HZO patients were vaccinated during the medical chart review since the varicella vaccine was introduced in 1995 and the zoster vaccine was introduced thereafter. Follow-up studies may need to investigate the relationship between vaccination and the recurrence or chronicity of HZO.

In conclusion, this study demonstrates clinical characteristics of HZO. This is the first study to analyze the relationship between the initial treatment period and recurrent HZO. Sex, age, and immune status did not affect the disease course; however, conjunctivitis, epithelial keratitis, and stromal keratitis could be risk factors for chronic HZO. Additionally, epithelial keratitis, stromal keratitis, and increased IOP raised the rate of recurrence. Therefore, clinicians must carefully look for these clinical manifestations.

HZO recurred after an average of 6.24 months and not many cases were observed beyond one year. Therefore, especially if patients have risk factors such as epithelial keratitis, stromal keratitis, or increased IOP, ophthalmologists need to follow up on the disease progression for at least one year.

## Figures and Tables

**Table 1:** Demographic and clinical characteristics of the study population.

Demographic	No. (%) Mean ± SD or Range
Sex	
Male	66 (50.77)
Female	64 (49.23)
Age at presentation (years)	
Mean	55.18 ± 17.66
Range	11–89
Immune status	
Immunocompetent	116 (89.23)
Immunocompromised	14 (10.77)
Treatment duration (days)	
Mean	82.22 ± 199.56
Range	3–1575
Chronic HZO	31 (23.85)
Acute HZO	99 (76.15)
Recurrent HZO	19 (14.62)
No recurrent HZO	111 (85.38)

Abbreviations: HZO = herpes zoster ophthalmicus; SD = standard deviation.

**Table 2:** Clinical manifestation of HZO (*n* = 130).

Clinical Manifestation	Frequency (%)
No eye involvement, only rash	26 (20)
Conjunctivitis	49 (37.69)
Epithelial keratitis	60 (46.15)
Stromal keratitis	10 (7.69)
Endothelial keratitis	15 (11.54)
Uveitis	8 (6.15)
Increased IOP	10 (7.7)
Cranial nerve palsy	2 (1.5)

Abbreviations: HZO = herpes zoster ophthalmicus; IOP = intraocular pressure. Note: Patients were counted more than once if they presented with multiple clinical findings of HZO.

**Table 3:** Results from univariate and multivariate analyses of factors associated with chronic HZO (*n* = 31).

	Univariate Analysis		Multivariate Analysis	
Variable	OR (95% CI)	*p* Value ^1^	OR (95% CI)	*p* Value ^1^
Age (years)	0.985 (0.963–1.018)	**0.1911**	0.963 (0.927–1.001)	0.0530
Sex	1.900 (0.834–4.331)	**0.1268**	1.248 (0.344–4.533)	0.7124
Immune status	0.500 (0.106–2.367)	0.3823		
Conjunctivitis	2.133 (0.941–4.839)	**0.0698**	8.698 (1.619–46.736)	**0.0154**
Epithelial keratitis	2.682 (1.160–6.200)	**0.0211**	41.495 (1.82–20.29)	**0.0005**
Stromal keratitis	40.070 (4.826–332.720)	**0.0006**	46.605 (2.597–836.357)	**0.0005**
Endotheliitis	13.062 (3.773–45.221)	**<0.0001**	3.171 (0.401–25.079)	0.0693
Uveitis	11.640 (2.214–61.193)	**0.0037**	6.519 (0.324–131.281)	0.3798
Increased IOP	16.869 (3.356–84.800)	**0.0006**	4.815 (0.437–53.019)	0.1940

Abbreviations: HZO = herpes zoster ophthalmicus; OR = odds ratio; CI = confidence interval; IOP = intraocular pressure. Variables with *p* < 0.20 were included in the multivariate analysis. Factors with statistical significance are shown in bold. ^1^ Logistic regression.

**Table 4:** Results from univariate and multivariate analyses of factors associated with recurrent HZO (*n* = 19).

	Univariate Analysis		Multivariate Analysis	
Variable	HR (95% CI)	*p* Value ^1^	HR (95% CI)	*p* Value ^1^
Age (years)	1.000 (0.974–1.025)	0.9723	1.027 (0.980–1.067)	0.1661
Sex	3.161(1.138–8.777)	**0.0272**	2.606 (0.690–9.837)	0.1576
Immune status	1.052 (0.243–4.556)	0.9455		
Conjunctivitis	2.321 (0.933–5.771)	**0.0700**	2.671 (0.654–10.910)	0.1713
Epithelial keratitis	2.876 (1.092–7.571)	**0.0324**	5.550 (1.207–29.993)	**0.0465**
Stromal keratitis	14.924 (5.460–40.795)	**<0.0001**	18.811 (2.929–120.824)	**0.0020**
Endotheliitis	5.638 (2.214–14.353)	**0.0003**	0.221 (0.039–1.246)	0.0871
Uveitis	5.441 (1782–16.612)	**0.0029**	1.484 (0.218–10.094)	0.6863
Increased IOP	14.925 (5.818–38.286)	**<0.0001**	7.306 (1.606–33.242)	**0.0101**
Primary Tx duration	1.003 (1.002–1.004)	**<0.0001**	0.999 (0.997–1.001)	0.4745
Chronic course	32.146 (3.473–297.543)	**0.0022**	34.405 (3.647–324.611)	**0.0020**

Abbreviations: HZO = herpes zoster ophthalmicus; HR = hazard ratio; CI = confidence interval; Tx = treatment duration; IOP = intraocular pressure. Variables with *p* < 0.20 were included in the multivariate analysis. Factors with statistical significance are shown in bold. ^1^ Cox proportional hazards regression.

**Table 5:** Clinical manifestation of recurrent herpes zoster ophthalmicus (*n* = 19).

Patient Number	Sex	Age (Years)	Immune Status	First Ocular Manifestation	Recurred Ocular Manifestation
1	F	40	competent	S,U	S
2	M	35	competent	S,I	S
3	F	22	competent	E	S
4	F	58	competent	C,E,I	C
5	M	76	competent	C,E,S,e	E,S,e
6	F	57	competent	E	S
7	F	54	competent	C,e	E
8	M	63	competent	E,S,I	S, I
9	F	61	competent	C,S,e,U	S,e
10	F	59	competent	C,E	S
11	F	62	compromised	C,E	S,e
12	F	71	competent	C,E,e	S
13	F	23	competent	E,S	S
14	F	44	compromised	C,E,S	E,S
15	M	53	competent	C	C
16	F	71	competent	E,e,U	E
17	F	62	competent	C,E	E
18	F	67	competent	C,E	E
19	M	70	competent	E	E

Abbreviations: C = conjunctival keratitis; E = epithelial keratitis; S = stromal keratitis; e = endothelial keratitis; U = uveitis, I = increased intraocular pressure. Note: Of the ocular manifestations of recurrent HZO, stromal keratitis was the most common clinical finding. Only two patients had an immunocompromised status. Three recurrent stromal keratitis patients did not have stromal keratitis at the initial visit.

## Data Availability

All datasets generated and/or analyzed during the current study are available from the corresponding author upon reasonable request.
